# Chiral crystallization of a zinc(II) complex

**DOI:** 10.1107/S2056989021003650

**Published:** 2021-04-23

**Authors:** Shabana Noor, Shintaro Suda, Tomoyuki Haraguchi, Fehmeeda Khatoon, Takashiro Akitsu

**Affiliations:** a Department of Applied Sciences & Humanities, Faculty of Engineering & Technology, Jamia Millia Islamia, New Delhi-110025, India; b Department of Chemistry, Faculty of Science, Tokyo University of Science, 1-3 Kagurazaka, Shinjuku-ku, Tokyo 162-8601, Japan; c Department of Applied, Sciences and Humanities , Faculty of Engineering and Technology, Jamia Millia Islamia, New Delhi - 110025, India

**Keywords:** chiral crystallization, zinc(II), Hirshfeld analysis, crystal structure

## Abstract

The compound, {6,6′-dimeth­oxy-2,2′-[(4-azaheptane-1,7-di­yl)bis­(nitrilo­meth­an­yl­idyne)]diphenolato}zinc(II) methanol monosolvate, [Zn(C_22_H_27_N_3_O_4_)]·CH_3_OH, at 298 K crystallizes in the ortho­rhom­bic space group P*na*2_1_. The Zn atom is coordinated by a penta­dentate Schiff base ligand in a distorted trigonal–bipyramidal N_3_O_2_ geometry. The equatorial plane is formed by the two phenolic O and one amine N atom. The axial positions are occupied by two amine N atoms. The distorted bipyramidal geometry is also supported by the trigonality index (τ), which is found to be 0.85 for the mol­ecule. In the crystal, methanol solvent mol­ecule is connected to the complex mol­ecule by an O—H⋯O hydrogen bond and the complex mol­ecules are connected by weak supra­molecular inter­actions, so achiral mol­ecules generate a chiral crystal. The Hirshfeld surface analysis suggests that H⋯H contacts account for the largest percentage of all inter­actions.

## Abstract 2

In this paper, we report on the chiral crystallization of achiral mol­ecules.

## Chemical context   

Schiff bases and their coordination compounds play an important role in metal coordination chemistry owing to their thermal stability, relevant biological properties and high synthesis flexibility (Bartyzel, 2018 ▸; Siddiqui *et al.*, 2006 ▸; Sacconi, 1966 ▸). These ligands are able to coordinate a wide variety of metal ions and to stabilize them in various oxidation states. The coordination geometry of the complexes depends upon the chemical structure of the chosen ligand, the coordination geometry preferred by the metal, the metal-to-ligand ratio, the reaction time and temperature (Thakurta *et al.*, 2010 ▸; Fleck *et al.*, 2013 ▸; Sanmartín *et al.*, 2001 ▸; Khalaji *et al.*, 2011 ▸). A number of zinc(II) complexes with different Schiff base ligands and their potential applications in sensing and as anti­bacterial and anti­cancer agents have been documented in the literature (Lui *et al.*,2019 ▸; Niu *et al.*, 2015 ▸; Tang *et al.*, 2013 ▸; AlDamen *et al.*, 2016 ▸; Iksi *et al.*, 2020 ▸). In addition, lanthanide Schiff base compounds are the subject of immense research inter­est because of their unique structures and their potential applications in advanced materials such as undoped semiconductors, magnetic, catalytic and florescent and non-linear optical materials (Li *et al.*, 2016 ▸; Ishikawa *et al.*, 2003 ▸; Long *et al.*, 2018*b* ▸).

In a previous work, we reported the crystal and mol­ecular structure of a Cu^II^ complex based on Schiff base ligand *N*1,*N*3-bis­(3-meth­oxy­salicylicyl­idene)di­ethyl­enetri­amine where two Schiff base ligands join two Cu^II^ ions in a chelate–spacer–chelate mode, in which the protonated aliphatic secondary amine moieties represents the spacer to form a double helix (Noor *et al.*, 2018 ▸). In an another report, we redetermined the crystal structure of an organic–inorganic salt of an Mn^II^–Schiff base ligand complex Mn(C_18_H_18_N_2_O_4_)(H_2_O)_2_]ClO_4_ at 100 K. In contrast to crystal-structure determinations at room temperature (Akitsu *et al.*, 2005 ▸, Bermejo *et al.*, 2007 ▸), positional disorder of the ethyl­ene bridge in the Schiff base and the perchlorate anion was not observed at 100 K (Noor *et al.* 2016 ▸). We now report the chiral crystallization on a zinc(II) complex.
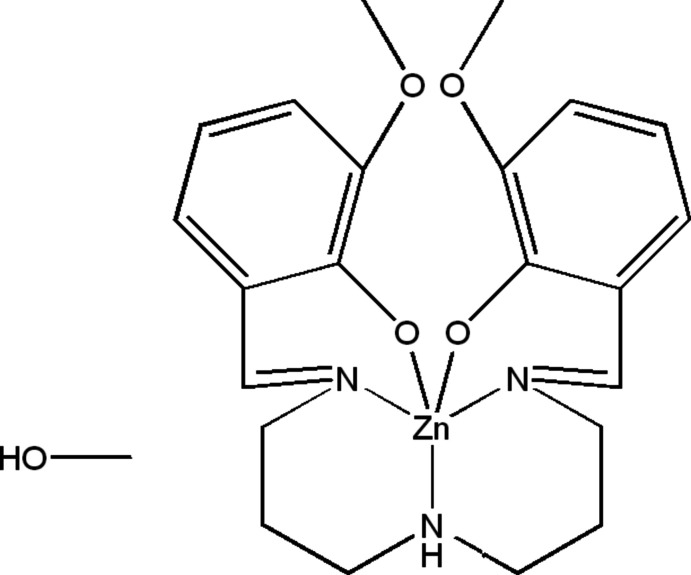


## Structural commentary   

**Figure 1:**
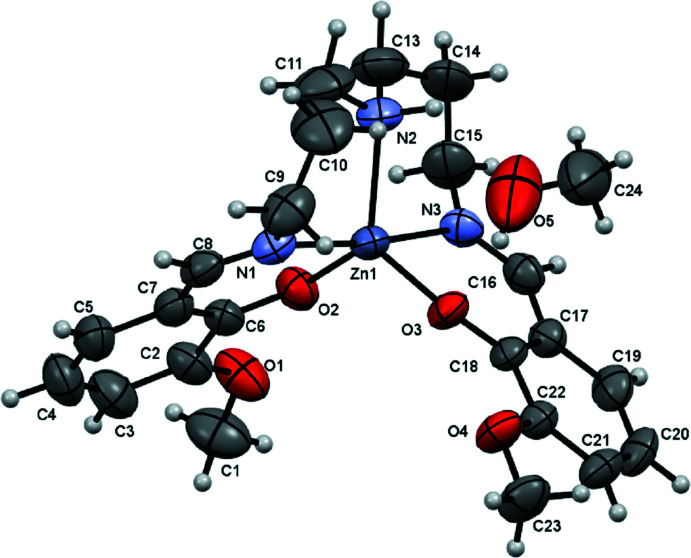
The mol­ecular structure of the title compound, showing the atom-labelling scheme.

In the title compound (Fig. 1 ▸), the Zn atom is coordinated by a penta­dentate Schiff base ligand in distorted trigonal–bipyramidal [ONNNO] geometry. The equatorial plane is formed by the two phenolic O [Zn—O3 = 2.001 (2) Å; Zn—O2 = 1.975 (2) Å] and one amine N [Zn—N2 = 2.152 (3) Å]. The axial positions are occupied by amine N atoms [Zn—N1 = 2.094 (2) Å, Zn—N3; 2.107 (2) Å]. The trigonality index (τ) for the complex is calculated as τ = (β − α) /60 where α and β are the main opposing angles in the coordination polyhedron (Addison *et al.*, 1984 ▸). For perfect square-pyramidal and trigonal–bipyramidal coordination geometries, the values of τ are zero and unity, respectively. In the present complex, for Zn, β = O2—Zn—O3 = 122.87 (10)° and α = N1—Zn—N3 = 173.91 (12)° so the trigonality index is 0.85. According to this value, the coordination geometry around the zinc ion is best described as distorted trigonal–bipyramidal. An intra­molecular O—H⋯O hydrogen bond is observed between the meth­oxy function and the oxygen atom in the six-membered ring (Table 1 ▸). This mol­ecule has neither an asymmetric carbon nor a helical structure, so it is an achiral compound.

## Supra­molecular features   

**Figure 2:**
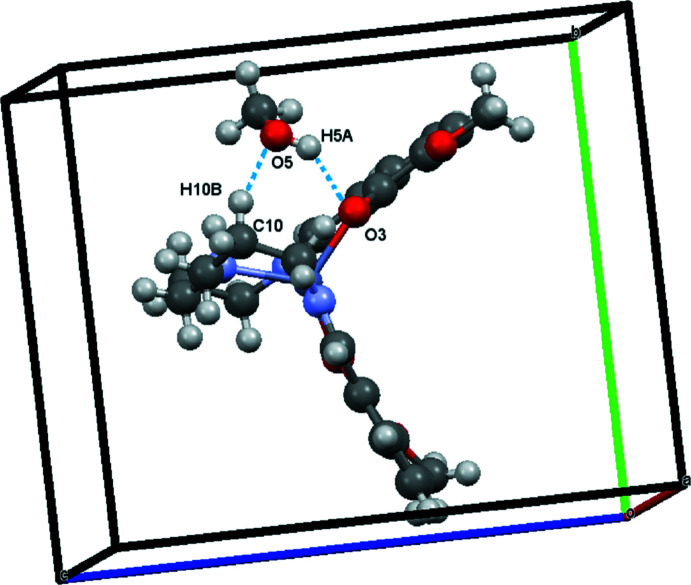
A view of the O5—H5*A*⋯O3 and C10—H10*B*⋯O5 inter­actions (dashed lines).

**Figure 3:**
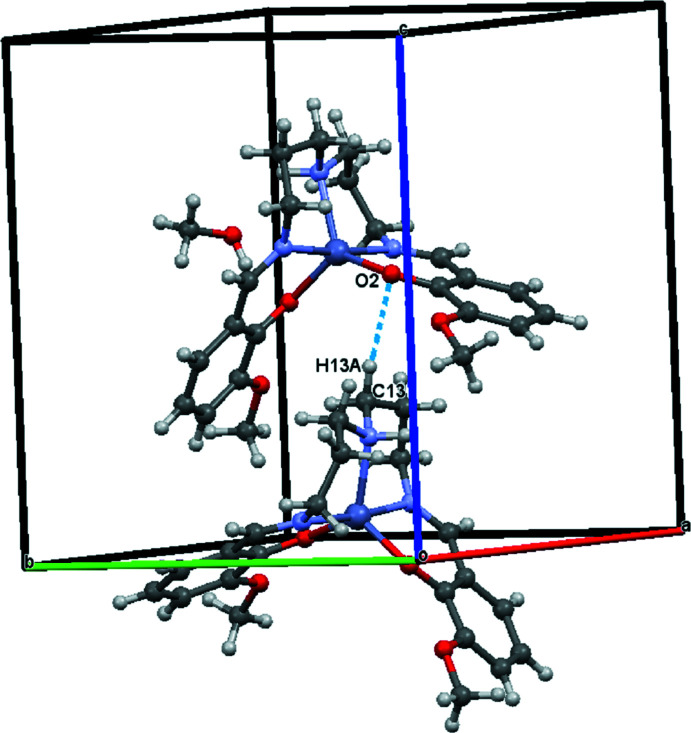
A view of the C13—H13*A*⋯O2 inter­action (dashed lines).

In the crystal, mol­ecules are connected by O—H⋯O hydrogen bonds (Fig. 2 ▸, Table 1 ▸). In addition, weak supra­molecular C—H⋯O inter­actions are found (Table 1 ▸ and Fig. 3 ▸).

## Hirshfeld Surface analysis   

**Figure 4:**
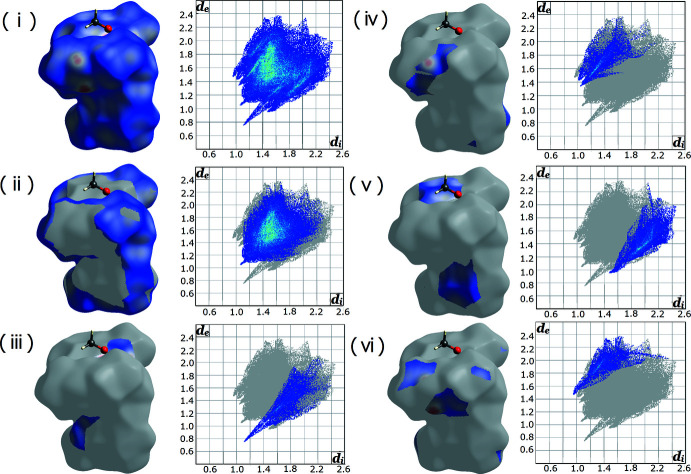
The three-dimensional Hirshfeld surface showing the inter­molecular inter­actions plotted over *d*_norm_ and the two-dimensional fingerprint plots of the title compound, showing (i) all inter­actions, and delineated into (ii) H⋯H, (iii) O⋯H, (iv) H⋯O, (v) C⋯H and (vi) H⋯C inter­actions.

In order to visualize the inter­molecular inter­actions in the crystal of the title compound, a Hirshfeld surface analysis (Spackman & Jayatilaka, 2009 ▸) was performed with *CrystalExplorer17.5* (Turner *et al.*, 2017 ▸). The fingerprint plot for this structure shows typical ‘wings’ (Fig. 4 ▸i). The percentage contribution to the Hirshfeld surface area by close contacts with H atoms inside the surface and H atoms outside is 57.4% (Fig. 4 ▸ii), for O atoms inside the surface and H atoms outside it is 9.1% (Fig. 4 ▸iii), for H atoms inside the surface and O atoms outside it is 8.5% (Fig. 4 ▸iv), for C atoms inside the surface and H atoms outside it is 14.5% (Fig. 4 ▸v), and for H atoms inside the surface and C atoms outside it is 8.2% (Fig. 4 ▸vi). Hirshfeld surface analysis of the H⋯O inter­action clearly shows the close inter­molecular contact near methanol, (*d*_i_ is 1.1 Å and *d*_e_ is 0.75 Å).

## Database survey   

A search in the Cambridge Structural Database (CSD, Version 5.41, update November 2019; Groom *et al.*, 2016 ▸) for similar structures returned three relevant entries: (2,2′-bi­pyridine-κ^2^ *N*,*N*′)[*N*-(2-oxido-1-naphthyl­idene)threoninato-κ^3^ *O* ^1^,*N*,*O* ^2^]copper(II) (BIZGIB; Qiu *et al.*, 2008 ▸), di­aqua­(*N*-salicyl­idene-l-threoninato)copper(II) (SLCDCU; Korhonen & Hämäläinen, 1981 ▸) and [*N*-(3-meth­oxy-2-oxido­benzyl­idene-κ*O* ^2^)threoninato-κ^2^ *O* ^1^,*N*](1,10-phenanthroline-κ^2^ *N*,*N*′)copper(II) hemihydrate (UQUYUB; Jing *et al.*, 2011 ▸). The metal atom in BIZGIB is five-coordinated by one N atom and two O atoms, and two N atoms from a distorted square-pyramidal 2,2-bi­pyridine ligand. In the crystal, a two-dimensional network is formed by a combination of inter­molecular O—H⋯O and C—H⋯O hydrogen bonds. In SLCDCU, two mol­ecules form square planes by two inter­molecular hydrogen bonds. In UQUYUB, inter­molecular O—H⋯O hydrogen bonds form a one-dimensional left-handed helical structure extending parallel to [001].

## Synthesis and crystallization   

The Schiff base ligand H_2_ *L* was prepared according to a reported procedure (Matar *et al.*, 2015 ▸) by a condensation reaction between 3-meth­oxy-2-hy­droxy­benzaldehyde (10 mmol, 1.52 mg) and di­propyl­enetri­amine (5.0 mmol, 0.641 mL) in ethanol solution (30 cm^3^) under reflux conditions. After solvent removal, a yellow oil was obtained in 85% yield. ν(C=N) 1630 cm^−1^.

The title compound was synthesized by the reaction of H_2_ *L* (1 mmol, 0.399 mg) with Zn(OAc)_2_·2H_2_O (1 mmol, 0.18 mg) in MeOH:H_2_O (*v*/*v*, 10:1) (50 cm^3^) with a few drops of LiOH (1%). The reaction mixture was heated to 343 K for 1 h. The yellow solid obtained was filtered off and dried. ν(C=N) 1618 cm^−1^. Single crystals suitable for X-ray crystallography were obtained several days after dissolving the solid in 40 cm^3^ of hot methanol.

## Refinement   

Crystal data, data collection and structure refinement details are summarized in Table 2 ▸. All C-bound H atoms were placed in geometrically calculated positions and refined using a riding model [C—H = 0.95 Å and *U*_iso_(H) = 1.2*U*_eq_(C) for aromatic H atoms, C—H = 0.98 Å and *U*_iso_(H) = 1.5*U*_eq_(C) for methyl H atoms]. The O-bound H atom was located in a difference-Fourier map and refined using a riding model [O—H = 0.82 Å and *U*_iso_(H) = 1.5*U*_eq_(O)].

## Supplementary Material

Crystal structure: contains datablock(s) 1R, I. DOI: 10.1107/S2056989021003650/jy2006sup1.cif

Structure factors: contains datablock(s) I. DOI: 10.1107/S2056989021003650/jy2006Isup2.hkl

CCDC reference: 2075457

Additional supporting information:  crystallographic information; 3D view; checkCIF report

## Figures and Tables

**Table 1:** Hydrogen-bond geometry (Å, °)

*D*—H⋯*A*	*D*—H	H⋯*A*	*D*⋯*A*	*D*—H⋯*A*
C13—H13*A*⋯O2^i^	0.97	2.58	3.549 (6)	172
C10—H10*B*⋯O5	0.97	2.50	3.340 (7)	144
O5—H5*A*⋯O3	0.82	2.01	2.722 (5)	144

Symmetry code: (i) -x+1, -y+1, z+{\script{1\over 2}}.

**Table 2:** Experimental details

Crystal data	
Chemical formula	[Zn(C_22_H_27_N_3_O_4_)]·CH_4_O
*M*_r_	494.88
Crystal system, space group	Orthorhombic, *P* *n* *a*2_1_
Temperature (K)	293
*a*, *b*, *c* (Å)	14.5937 (6), 11.4425 (5), 13.5794 (5)
*V* (Å^3^)	2267.60 (16)
*Z*	4
Radiation type	Mo *K*α
μ (mm^−1^)	1.12
Crystal size (mm)	0.53 × 0.49 × 0.45

Data collection	
Diffractometer	Bruker APEXIII CCD
Absorption correction	Multi-scan (*SADABS*; Bruker, 2017 ▸)
*T*_min_, *T*_max_	0.53, 0.63
No. of measured, independent and observed [*I* > 2σ(*I*)] reflections	28025, 6099, 4682
*R*_int_	0.035
(sin θ/λ)_max_ (Å^−1^)	0.730

Refinement	
*R*[*F* ^2^ > 2σ(*F* ^2^)], *wR*(*F* ^2^), *S*	0.035, 0.087, 1.04
No. of reflections	6099
No. of parameters	294
No. of restraints	1
H-atom treatment	H-atom parameters constrained
Δρ_max_, Δρ_min_ (e Å^−3^)	0.56, −0.47
Absolute structure	Flack *x* determined using 1852 quotients [(*I* ^+^)−(*I* ^−^)]/[(*I* ^+^)+(*I* ^−^)] (Parsons *et al.*, 2013 ▸)
Absolute structure parameter	0.006 (5)

Computer programs: *APEX3* and *SAINT* (Bruker, 2017 ▸), *SHELXT2014/5* (Sheldrick, 2015*a* ▸), *SHELXL2016/6* (Sheldrick, 2015*b* ▸), *shelXle* (Hübschle *et al.*, 2011 ▸) and *SHELXTL* (Sheldrick, 2008 ▸).

## supplementary crystallographic information

### Crystal data

**Table:**

[Zn(C_22_H_27_N_3_O_4_)]·CH_4_O	*D*_x_ = 1.450 Mg m^−^^3^
*M**_r_* = 494.88	Mo *K*α radiation, λ = 0.71073 Å
Orthorhombic, *P**n**a*2_1_	Cell parameters from 3270 reflections
*a* = 14.5937 (6) Å	θ = 2.2–23.9°
*b* = 11.4425 (5) Å	µ = 1.12 mm^−^^1^
*c* = 13.5794 (5) Å	*T* = 293 K
*V* = 2267.60 (16) Å^3^	Prism, yellow
*Z* = 4	0.53 × 0.49 × 0.45 mm
*F*(000) = 1040	

### Data collection

**Table:**

Bruker APEXIII CCD diffractometer	4682 reflections with *I* > 2σ(*I*)
Detector resolution: 7.3910 pixels mm^-1^	*R*_int_ = 0.035
φ and ω scans	θ_max_ = 31.2°, θ_min_ = 2.3°
Absorption correction: multi-scan (SADABS; Bruker, 2017)	*h* = −21→20
*T*_min_ = 0.53, *T*_max_ = 0.63	*k* = −16→16
28025 measured reflections	*l* = −18→18
6099 independent reflections	

### Refinement

**Table:**

Refinement on *F*^2^	H-atom parameters constrained
Least-squares matrix: full	*w* = 1/[σ^2^(*F*_o_^2^) + (0.0516*P*)^2^] where *P* = (*F*_o_^2^ + 2*F*_c_^2^)/3
*R*[*F*^2^ > 2σ(*F*^2^)] = 0.035	(Δ/σ)_max_ = 0.001
*wR*(*F*^2^) = 0.087	Δρ_max_ = 0.56 e Å^−^^3^
*S* = 1.04	Δρ_min_ = −0.47 e Å^−^^3^
6099 reflections	Extinction correction: SHELXL-2016/6 (Sheldrick 2015b)
294 parameters	Extinction coefficient: 0.0091 (12)
1 restraint	Absolute structure: Flack *x* determined using 1852 quotients [(*I*^+^)-(*I*^-^)]/[(*I*^+^)+(*I*^-^)] (Parsons *et al.*, 2013)
Primary atom site location: dual	Absolute structure parameter: 0.006 (5)
Hydrogen site location: inferred from neighbouring sites	

### Special details

**Table:**

Geometry. All esds (except the esd in the dihedral angle between two l.s. planes) are estimated using the full covariance matrix. The cell esds are taken into account individually in the estimation of esds in distances, angles and torsion angles; correlations between esds in cell parameters are only used when they are defined by crystal symmetry. An approximate (isotropic) treatment of cell esds is used for estimating esds involving l.s. planes.
Refinement. Reflections were merged by SHELXL according to the crystal class for the calculation of statistics and refinement. _reflns_Friedel_fraction is defined as the number of unique Friedel pairs measured divided by the number that would be possible theoretically, ignoring centric projections and systematic absences.

### Fractional atomic coordinates and isotropic or equivalent isotropic displacement parameters (Å^2^)

**Table:**

	*x*	*y*	*z*	*U*_iso_*/*U*_eq_	
Zn1	0.52170 (2)	0.52816 (3)	0.56294 (4)	0.04284 (12)	
O1	0.40493 (18)	0.1936 (2)	0.4333 (2)	0.0724 (8)	
N1	0.65984 (15)	0.4787 (2)	0.5612 (3)	0.0493 (5)	
C1	0.3627 (3)	0.1110 (5)	0.3702 (4)	0.0857 (13)	
H1A	0.396883	0.10538	0.310017	0.129*	
H1B	0.301217	0.135516	0.355979	0.129*	
H1C	0.361408	0.036045	0.401999	0.129*	
O2	0.48708 (13)	0.3687 (2)	0.52144 (19)	0.0520 (5)	
N2	0.5413 (2)	0.5782 (3)	0.7143 (2)	0.0650 (8)	
H2	0.529572	0.662482	0.715739	0.078*	
C2	0.4980 (2)	0.1835 (3)	0.4463 (3)	0.0549 (8)	
O3	0.53164 (12)	0.6620 (2)	0.46866 (16)	0.0457 (5)	
N3	0.38225 (17)	0.5711 (2)	0.58014 (19)	0.0486 (6)	
C3	0.5487 (3)	0.0903 (3)	0.4162 (3)	0.0693 (10)	
H3	0.520034	0.028129	0.38465	0.083*	
C4	0.6437 (3)	0.0868 (3)	0.4321 (3)	0.0720 (10)	
H4	0.677466	0.021275	0.414127	0.086*	
O4	0.58178 (14)	0.7754 (2)	0.30994 (16)	0.0545 (5)	
C5	0.6856 (2)	0.1802 (3)	0.4741 (3)	0.0610 (8)	
H5	0.74875	0.179029	0.483288	0.073*	
O5	0.5841 (3)	0.8341 (4)	0.5950 (4)	0.140 (2)	
H5A	0.581512	0.801846	0.541104	0.21*	
C6	0.5391 (2)	0.2829 (3)	0.4930 (2)	0.0453 (6)	
C7	0.6353 (2)	0.2785 (3)	0.5037 (2)	0.0479 (6)	
C8	0.68830 (19)	0.3763 (3)	0.54147 (19)	0.0502 (7)	
H8	0.750228	0.362278	0.552506	0.06*	
C9	0.7273 (3)	0.5641 (4)	0.5954 (3)	0.0735 (11)	
H9A	0.731688	0.625957	0.54678	0.088*	
H9B	0.786656	0.526017	0.59861	0.088*	
C10	0.7079 (3)	0.6176 (5)	0.6924 (4)	0.0995 (17)	
H10A	0.763926	0.615887	0.730831	0.119*	
H10B	0.692303	0.698978	0.681751	0.119*	
C11	0.6358 (4)	0.5649 (5)	0.7506 (3)	0.0890 (14)	
H11A	0.638748	0.597758	0.816366	0.107*	
H11B	0.648732	0.48201	0.756321	0.107*	
C13	0.4737 (4)	0.5284 (4)	0.7786 (4)	0.0809 (15)	
H13A	0.488874	0.551024	0.845482	0.097*	
H13B	0.47908	0.444052	0.774871	0.097*	
C14	0.3754 (4)	0.5598 (5)	0.7606 (3)	0.0848 (14)	
H14A	0.369652	0.644202	0.763231	0.102*	
H14B	0.338813	0.527906	0.81384	0.102*	
C15	0.3349 (3)	0.5172 (4)	0.6626 (3)	0.0708 (11)	
H15A	0.340711	0.432901	0.658309	0.085*	
H15B	0.270299	0.536678	0.659841	0.085*	
C16	0.33585 (19)	0.6329 (3)	0.5196 (2)	0.0491 (7)	
H16	0.273212	0.638729	0.531279	0.059*	
C17	0.37116 (18)	0.6945 (3)	0.4351 (2)	0.0444 (6)	
C18	0.46571 (18)	0.7052 (3)	0.4135 (2)	0.0395 (6)	
C19	0.3057 (2)	0.7452 (3)	0.3712 (3)	0.0560 (8)	
H19	0.243749	0.739602	0.386674	0.067*	
C20	0.3314 (2)	0.8021 (3)	0.2878 (3)	0.0606 (8)	
H20	0.287289	0.833751	0.246236	0.073*	
C21	0.4235 (2)	0.8127 (3)	0.2647 (2)	0.0536 (8)	
H21	0.440955	0.850523	0.207094	0.064*	
C22	0.4898 (2)	0.7674 (3)	0.3267 (2)	0.0432 (6)	
C23	0.6092 (3)	0.8324 (4)	0.2225 (3)	0.0684 (10)	
H23A	0.585095	0.791083	0.166649	0.103*	
H23B	0.674874	0.833728	0.218869	0.103*	
H23C	0.586221	0.910978	0.222502	0.103*	
C24	0.5060 (6)	0.8915 (6)	0.6115 (6)	0.123 (2)	
H24A	0.519332	0.971869	0.626096	0.185*	
H24B	0.47469	0.856789	0.666377	0.185*	
H24C	0.467835	0.887306	0.554073	0.185*	

### Atomic displacement parameters (Å^2^)

**Table:**

	*U*^11^	*U*^22^	*U*^33^	*U*^12^	*U*^13^	*U*^23^
Zn1	0.04857 (18)	0.04890 (19)	0.03106 (16)	0.00482 (12)	−0.00223 (18)	0.00193 (18)
O1	0.0517 (13)	0.0781 (16)	0.0876 (19)	−0.0119 (13)	0.0060 (13)	−0.0318 (16)
N1	0.0489 (10)	0.0563 (13)	0.0427 (11)	−0.0010 (10)	−0.0077 (17)	0.0044 (15)
C1	0.075 (3)	0.101 (3)	0.081 (3)	−0.025 (2)	0.002 (2)	−0.032 (3)
O2	0.0430 (9)	0.0563 (12)	0.0568 (13)	0.0031 (9)	0.0028 (9)	−0.0092 (11)
N2	0.0852 (19)	0.073 (2)	0.0362 (14)	0.0177 (17)	−0.0110 (14)	−0.0066 (15)
C2	0.0546 (15)	0.057 (2)	0.0533 (19)	−0.0019 (15)	0.0102 (15)	−0.0081 (17)
O3	0.0393 (9)	0.0580 (13)	0.0399 (10)	0.0031 (9)	−0.0055 (8)	0.0109 (10)
N3	0.0498 (12)	0.0543 (13)	0.0416 (15)	0.0015 (11)	0.0116 (11)	0.0017 (11)
C3	0.078 (2)	0.056 (2)	0.073 (2)	−0.0023 (18)	0.011 (2)	−0.0154 (19)
C4	0.075 (2)	0.058 (2)	0.083 (3)	0.0167 (18)	0.013 (2)	−0.010 (2)
O4	0.0462 (10)	0.0713 (14)	0.0459 (11)	−0.0025 (10)	−0.0006 (9)	0.0192 (11)
C5	0.0577 (17)	0.065 (2)	0.0597 (19)	0.0159 (16)	0.0042 (15)	0.0035 (17)
O5	0.152 (4)	0.088 (2)	0.181 (5)	0.016 (2)	−0.098 (4)	−0.033 (3)
C6	0.0496 (14)	0.0481 (16)	0.0383 (14)	0.0045 (13)	0.0059 (12)	−0.0011 (13)
C7	0.0490 (14)	0.0536 (16)	0.0411 (15)	0.0061 (14)	0.0025 (13)	0.0063 (13)
C8	0.0425 (12)	0.0681 (18)	0.0399 (18)	0.0039 (13)	−0.0036 (11)	0.0076 (13)
C9	0.068 (2)	0.072 (2)	0.080 (3)	−0.0142 (19)	−0.0219 (19)	0.007 (2)
C10	0.085 (3)	0.116 (4)	0.097 (4)	−0.006 (3)	−0.036 (3)	−0.028 (3)
C11	0.111 (4)	0.109 (3)	0.047 (2)	−0.009 (3)	−0.029 (2)	−0.002 (2)
C13	0.115 (4)	0.088 (4)	0.041 (2)	0.000 (2)	0.009 (2)	0.0057 (19)
C14	0.108 (3)	0.099 (3)	0.047 (2)	0.027 (3)	0.028 (2)	0.010 (2)
C15	0.074 (2)	0.075 (2)	0.063 (2)	−0.0035 (19)	0.026 (2)	0.0137 (19)
C16	0.0394 (12)	0.0556 (16)	0.0522 (15)	0.0033 (13)	0.0045 (13)	−0.0066 (15)
C17	0.0406 (13)	0.0490 (14)	0.0436 (14)	0.0035 (12)	−0.0033 (12)	−0.0017 (13)
C18	0.0405 (12)	0.0432 (14)	0.0347 (13)	0.0033 (11)	−0.0082 (10)	0.0015 (11)
C19	0.0407 (14)	0.0634 (19)	0.064 (2)	0.0070 (14)	−0.0124 (15)	−0.0038 (17)
C20	0.0544 (16)	0.0613 (19)	0.066 (2)	0.0089 (15)	−0.0237 (16)	0.0129 (17)
C21	0.0617 (18)	0.0544 (17)	0.0446 (16)	−0.0014 (15)	−0.0120 (15)	0.0125 (14)
C22	0.0459 (14)	0.0437 (15)	0.0401 (15)	−0.0009 (12)	−0.0065 (12)	0.0036 (13)
C23	0.064 (2)	0.083 (3)	0.058 (2)	−0.002 (2)	0.0065 (18)	0.026 (2)
C24	0.189 (6)	0.083 (4)	0.097 (4)	0.033 (4)	−0.034 (4)	−0.016 (3)

### Geometric parameters (Å, º)

**Table:**

Zn1—O2	1.975 (2)	C9—C10	1.480 (7)
Zn1—O3	2.001 (2)	C9—H9A	0.97
Zn1—N1	2.094 (2)	C9—H9B	0.97
Zn1—N3	2.107 (2)	C10—C11	1.448 (7)
Zn1—N2	2.152 (3)	C10—H10A	0.97
O1—C2	1.375 (4)	C10—H10B	0.97
O1—C1	1.416 (5)	C11—H11A	0.97
N1—C8	1.273 (4)	C11—H11B	0.97
N1—C9	1.462 (5)	C13—C14	1.499 (7)
C1—H1A	0.96	C13—H13A	0.97
C1—H1B	0.96	C13—H13B	0.97
C1—H1C	0.96	C14—C15	1.535 (7)
O2—C6	1.300 (4)	C14—H14A	0.97
N2—C13	1.435 (6)	C14—H14B	0.97
N2—C11	1.473 (6)	C15—H15A	0.97
N2—H2	0.98	C15—H15B	0.97
C2—C3	1.361 (5)	C16—C17	1.443 (4)
C2—C6	1.432 (5)	C16—H16	0.93
O3—C18	1.316 (3)	C17—C19	1.415 (4)
N3—C16	1.278 (4)	C17—C18	1.416 (4)
N3—C15	1.453 (4)	C18—C22	1.421 (4)
C3—C4	1.403 (6)	C19—C20	1.359 (5)
C3—H3	0.93	C19—H19	0.93
C4—C5	1.357 (6)	C20—C21	1.386 (5)
C4—H4	0.93	C20—H20	0.93
O4—C22	1.365 (4)	C21—C22	1.382 (4)
O4—C23	1.413 (4)	C21—H21	0.93
C5—C7	1.402 (4)	C23—H23A	0.96
C5—H5	0.93	C23—H23B	0.96
O5—C24	1.334 (8)	C23—H23C	0.96
O5—H5A	0.82	C24—H24A	0.96
C6—C7	1.413 (4)	C24—H24B	0.96
C7—C8	1.453 (5)	C24—H24C	0.96
C8—H8	0.93		
			
O2—Zn1—O3	122.87 (10)	C9—C10—H10A	108.1
O2—Zn1—N1	89.63 (9)	C11—C10—H10B	108.1
O3—Zn1—N1	97.45 (10)	C9—C10—H10B	108.1
O2—Zn1—N3	90.01 (9)	H10A—C10—H10B	107.3
O3—Zn1—N3	87.84 (9)	C10—C11—N2	117.1 (4)
N1—Zn1—N3	173.91 (12)	C10—C11—H11A	108.0
O2—Zn1—N2	123.50 (13)	N2—C11—H11A	108.0
O3—Zn1—N2	113.44 (12)	C10—C11—H11B	108.0
N1—Zn1—N2	87.40 (13)	N2—C11—H11B	108.0
N3—Zn1—N2	87.73 (11)	H11A—C11—H11B	107.3
C2—O1—C1	116.9 (3)	N2—C13—C14	117.6 (4)
C8—N1—C9	117.5 (3)	N2—C13—H13A	107.9
C8—N1—Zn1	124.4 (2)	C14—C13—H13A	107.9
C9—N1—Zn1	117.7 (2)	N2—C13—H13B	107.9
O1—C1—H1A	109.5	C14—C13—H13B	107.9
O1—C1—H1B	109.5	H13A—C13—H13B	107.2
H1A—C1—H1B	109.5	C13—C14—C15	115.7 (4)
O1—C1—H1C	109.5	C13—C14—H14A	108.4
H1A—C1—H1C	109.5	C15—C14—H14A	108.4
H1B—C1—H1C	109.5	C13—C14—H14B	108.4
C6—O2—Zn1	129.32 (19)	C15—C14—H14B	108.4
C13—N2—C11	113.5 (4)	H14A—C14—H14B	107.4
C13—N2—Zn1	112.6 (3)	N3—C15—C14	110.5 (3)
C11—N2—Zn1	114.6 (3)	N3—C15—H15A	109.5
C13—N2—H2	105.0	C14—C15—H15A	109.5
C11—N2—H2	105.0	N3—C15—H15B	109.5
Zn1—N2—H2	105.0	C14—C15—H15B	109.5
C3—C2—O1	124.3 (4)	H15A—C15—H15B	108.1
C3—C2—C6	121.8 (3)	N3—C16—C17	126.3 (3)
O1—C2—C6	113.8 (3)	N3—C16—H16	116.8
C18—O3—Zn1	126.77 (18)	C17—C16—H16	116.8
C16—N3—C15	118.6 (3)	C19—C17—C18	119.8 (3)
C16—N3—Zn1	124.7 (2)	C19—C17—C16	116.5 (3)
C15—N3—Zn1	116.4 (2)	C18—C17—C16	123.7 (3)
C2—C3—C4	120.9 (4)	O3—C18—C17	124.3 (3)
C2—C3—H3	119.6	O3—C18—C22	118.7 (2)
C4—C3—H3	119.6	C17—C18—C22	117.1 (2)
C5—C4—C3	119.2 (3)	C20—C19—C17	121.3 (3)
C5—C4—H4	120.4	C20—C19—H19	119.3
C3—C4—H4	120.4	C17—C19—H19	119.3
C22—O4—C23	116.7 (2)	C19—C20—C21	119.9 (3)
C4—C5—C7	121.1 (3)	C19—C20—H20	120.0
C4—C5—H5	119.5	C21—C20—H20	120.0
C7—C5—H5	119.5	C22—C21—C20	120.5 (3)
C24—O5—H5A	109.5	C22—C21—H21	119.7
O2—C6—C7	125.2 (3)	C20—C21—H21	119.7
O2—C6—C2	119.2 (3)	O4—C22—C21	124.1 (3)
C7—C6—C2	115.7 (3)	O4—C22—C18	114.5 (2)
C5—C7—C6	121.3 (3)	C21—C22—C18	121.4 (3)
C5—C7—C8	116.1 (3)	O4—C23—H23A	109.5
C6—C7—C8	122.6 (3)	O4—C23—H23B	109.5
N1—C8—C7	127.6 (3)	H23A—C23—H23B	109.5
N1—C8—H8	116.2	O4—C23—H23C	109.5
C7—C8—H8	116.2	H23A—C23—H23C	109.5
N1—C9—C10	115.4 (4)	H23B—C23—H23C	109.5
N1—C9—H9A	108.4	O5—C24—H24A	109.5
C10—C9—H9A	108.4	O5—C24—H24B	109.5
N1—C9—H9B	108.4	H24A—C24—H24B	109.5
C10—C9—H9B	108.4	O5—C24—H24C	109.5
H9A—C9—H9B	107.5	H24A—C24—H24C	109.5
C11—C10—C9	116.9 (4)	H24B—C24—H24C	109.5
C11—C10—H10A	108.1		

### Hydrogen-bond geometry (Å, º)

**Table:**

*D*—H···*A*	*D*—H	H···*A*	*D*···*A*	*D*—H···*A*
C13—H13*A*···O2^i^	0.97	2.58	3.549 (6)	172
C10—H10*B*···O5	0.97	2.50	3.340 (7)	144
O5—H5*A*···O3	0.82	2.01	2.722 (5)	144

Symmetry code: (i) −*x*+1, −*y*+1, *z*+1/2.
